# Dihydroaustrasulfone Alcohol Inhibits PDGF-Induced Proliferation and Migration of Human Aortic Smooth Muscle Cells through Inhibition of the Cell Cycle

**DOI:** 10.3390/md13042390

**Published:** 2015-04-17

**Authors:** Yao-Chang Chen, Zhi-Hong Wen, Yen-Hsien Lee, Chu-Lun Chen, Han-Chun Hung, Chun-Hong Chen, Wu-Fu Chen, Min-Chien Tsai

**Affiliations:** 1 Department of Marine Biotechnology and Resources, National Sun Yat-sen University, Lienhai Road, Kaohsiung 804, Taiwan; E-Mail: bme02@ndmctsgh.edu.tw; 2 Department of Biomedical Engineering, National Defense Medical Center, Sec. 6, Minquan E. Road, Taipei 11490, Taiwan; 3 Doctoral Degree Program in Marine Biotechnology, National Sun Yat-sen University and Academia Sinica, Kaohsiung 80424, Taiwan; E-Mails: wzh@mail.nsysu.edu.tw (Z.-H.W.); hanchun25@gmail.com (H.-C.H.); anubis0620@gmail.com (C.-H.C.); 4 Graduate Institute of Medical Sciences, College of Medicine, Taipei Medical University, 250 Wuxing Street, Taipei 11042, Taiwan; E-Mail: edward0730@gmail.com; 5 Department of Physiology and Biophysics; Graduate Institute of Physiology, National Defense Medical Center, Sec. 6, Minquan E. Road, Taipei 11490, Taiwan; E-Mail: helenaprey@gmail.com; 6 Department of Neurosurgery, Kaohsiung Chang Gung Memorial Hospital and Chang Gung University College of Medicine, Kaohsiung 83301, Taiwan; E-Mail: ma4949@cgmh.org.tw

**Keywords:** dihydroaustrasulfone alcohol, vascular smooth muscle cell, proliferation, migration, phenotypic modulation, cell cycle, restenosis

## Abstract

Dihydroaustrasulfone alcohol is the synthetic precursor of austrasulfone, which is a marine natural product, isolated from the Taiwanese soft coral *Cladiella australis.* Dihydroaustrasulfone alcohol has anti-inflammatory, neuroprotective, antitumor and anti-atherogenic properties. Although dihydroaustrasulfone alcohol has been shown to inhibit neointima formation, its effect on human vascular smooth muscle cells (VSMCs) has not been elucidated. We examined the effects and the mechanisms of action of dihydroaustrasulfone alcohol on proliferation, migration and phenotypic modulation of human aortic smooth muscle cells (HASMCs). Dihydroaustrasulfone alcohol significantly inhibited proliferation, DNA synthesis and migration of HASMCs, without inducing cell death. Dihydroaustrasulfone alcohol also inhibited platelet-derived growth factor (PDGF)-induced expression of cyclin-dependent kinases (CDK) 2, CDK4, cyclin D1 and cyclin E. In addition, dihydroaustrasulfone alcohol inhibited PDGF-induced phosphorylation of extracellular signal-regulated kinase 1/2 (ERK1/2), whereas it had no effect on the phosphorylation of phosphatidylinositol 3-kinase (PI3K)/(Akt). Moreover, treatment with PD98059, a highly selective ERK inhibitor, blocked PDGF-induced upregulation of cyclin D1 and cyclin E and downregulation of p27^kip1^. Furthermore, dihydroaustrasulfone alcohol also inhibits VSMC synthetic phenotype formation induced by PDGF. For *in vivo* studies, dihydroaustrasulfone alcohol decreased smooth muscle cell proliferation in a rat model of restenosis induced by balloon injury. Immunohistochemical staining showed that dihydroaustrasulfone alcohol noticeably decreased the expression of proliferating cell nuclear antigen (PCNA) and altered VSMC phenotype from a synthetic to contractile state. Our findings provide important insights into the mechanisms underlying the vasoprotective actions of dihydroaustrasulfone alcohol and suggest that it may be a useful therapeutic agent for the treatment of vascular occlusive disease.

## 1. Introduction

Vascular smooth muscle cells (VSMCs) play a critical role in the development of vascular disease. The abnormal proliferation and migration of VSMCs has a pivotal role in the progression of vascular occlusion diseases, such as atherosclerosis and restenosis [1]. During vascular lesion formation, VSMCs change from their physiological contractile phenotype to the pathophysiological synthetic phenotype and migrate to the intima [2]. VSMCs in the media layers of the vessel walls of mature arteries maintain a low proliferation, contractile phenotype characterized by the low expression of proinflammatory factors, proliferation marker protein proliferating cell nuclear antigen (PCNA) and extracellular matrix (ECM), as well as high expression of contractile marker proteins, such as smooth muscle (SM)-α-actin and calponin [2]. The synthetic phenotype of VSMCs also contributes to the progression of atherosclerosis and the development of restenosis [2,3].

The formation and progression of vascular occlusion diseases are stimulated by various growth factors and cytokines, such as platelet-derived growth factor (PDGF). PDGF is one of the growth factors released by injured endothelial cells and smooth muscle cells after angioplasty or atherosclerosis [4,5]. PDGF has been reported as the most potent chemoattractant and regulator of the behavior of VSMCs [6,7,8].

Taiwan is surrounded by the sea, and natural products isolated from Taiwanese soft corals have been reported to have broad-spectrum biological effects, including anti-inflammatory, anti-neuroinflammatory and neuroprotective [9,10]. Dihydroaustrasulfone alcohol is the synthetic and bioactive precursor of austrasulfone, which is a marine natural product, isolated from the Formosa soft coral, *Cladiella australis* [11]. Previous studies have shown that dihydroaustrasulfone alcohol has therapeutic properties, such as anti-inflammatory, neuroprotective, anti-nociceptive, treatment of multiple sclerosis, anti-atherogenic and anti-tumor [11,12]. The inhibitory effects of dihydroaustrasulfone alcohol on the proinflammatory inducible nitric oxide synthase (iNOS) and cyclooxygenase-2 (COX-2) protein expression have been shown in LPS-stimulated macrophages and on neointima formation *in vivo* [11]. Neointima formation is due to abnormal VSMCs proliferation and migration from the media to the intimal layer during atherosclerosis and post-angioplasty restenosis. To date, restenosis is still a serious clinical problem [3,13,14,15]. Recent studies show that dihydroaustrasulfone alcohol may possess potential therapeutic properties. However, the effects of dihydroaustrasulfone alcohol on VSMCs have not been studied. Therefore, the effect of dihydroaustrasulfone alcohol on VSMCs should be explored, to examine its potential therapeutic role in atherosclerosis and restenosis. The purpose of the present investigation was to determine the effects of dihydroaustrasulfone alcohol on the proliferation, migration and phenotypic modulation of human VSMCs and to attempt to elucidate the mechanisms underlying these effects.

## 2. Results

### 2.1. Dihydroaustrasulfone Alcohol Inhibits PDGF-Stimulated Proliferation in Human Aortic Smooth Muscle Cells

**Figure 1:**
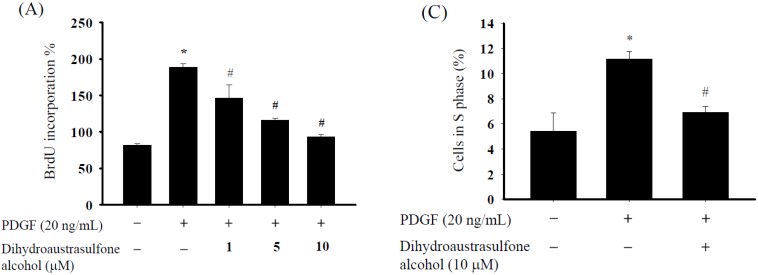

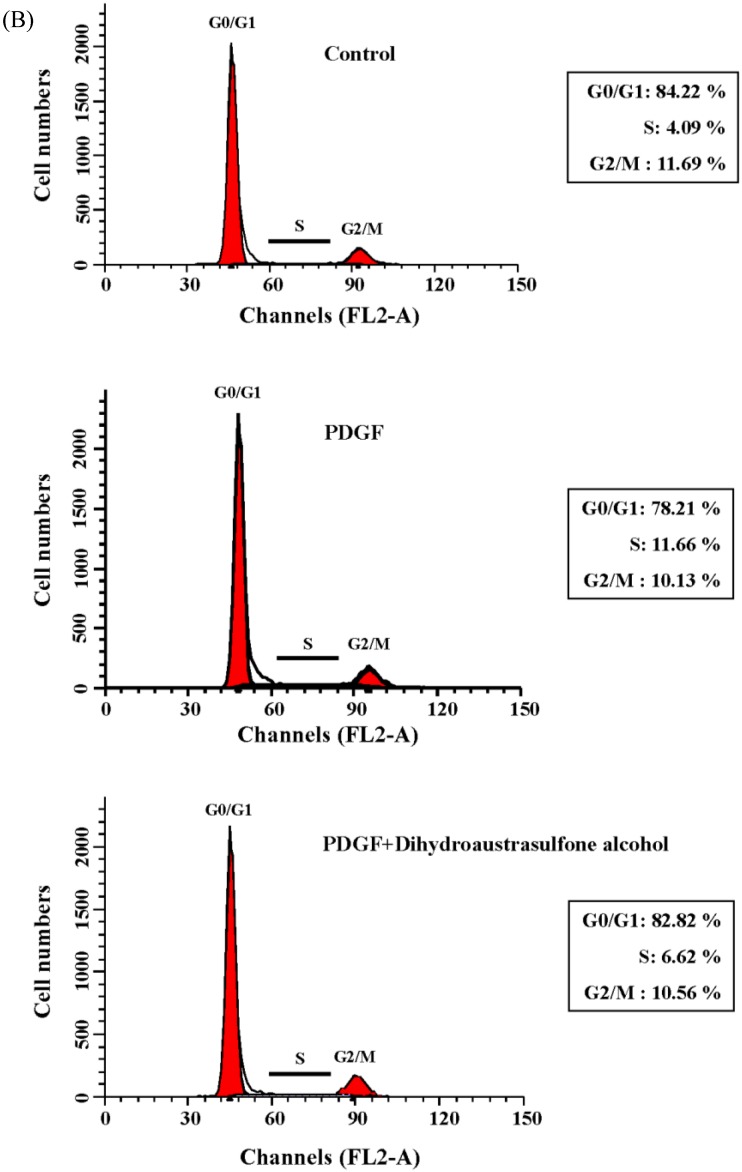
Effects of dihydroaustrasulfone alcohol on the proliferation of human aortic smooth muscle cells (HASMCs). (**A**) Dihydroaustrasulfone alcohol inhibits PDGF-stimulated DNA synthesis in HASMCs. HASMCs were serum-starved for 24 h and then preincubated with 1, 5 or 10 µM dihydroaustrasulfone alcohol for 1 h prior to the addition of PDGF (20 ng/mL) and incubation for 24 h. DNA synthesis was measured by using the BrdU incorporation assay; (**B**) Cell cycle distribution of HASMCs pretreated with dihydroaustrasulfone alcohol (10 μM) for 1 h before treatment with PDGF (20 ng/mL) and further incubation for 24 h. Cells were then stained with propidium iodide and analyzed for DNA content by using flow cytometry; (**C**) Quantitation of the percentage of cells in the S phase. Data are expressed as the mean ± SEM of three independent experiments. * *p* < 0.05 *versus* control, ^#^ *p* < 0.05 *versus* PDGF alone.

The bromodeoxyuridine (BrdU) incorporation assays and flow cytometry were used to examine the effects of various concentrations of dihydroaustrasulfone alcohol on the proliferation of HASMCs. The incorporation of the thymidine analog BrdU was measured to determine the effects of dihydroaustrasulfone alcohol on DNA synthesis. The HASMCs were pretreated with dihydroaustrasulfone alcohol (1, 5 or 10 µM) for 1 h, followed by the addition of PDGF (20 ng/mL). Dihydroaustrasulfone alcohol pretreatment significantly inhibited PDGF-induced DNA synthesis dose-dependently (Figure 1A). The half-maximal inhibitory concentration (IC_50_) was 9.4 μM. In the cell cycle analysis, PDGF induced significant S phase transition compared with controls, and this was significantly suppressed by pretreatment with 10 µM dihydroaustrasulfone alcohol (Figure 1B,C).

### 2.2. Dihydroaustrasulfone Alcohol Does not Affect HASMCs Viability

**Figure 2:**
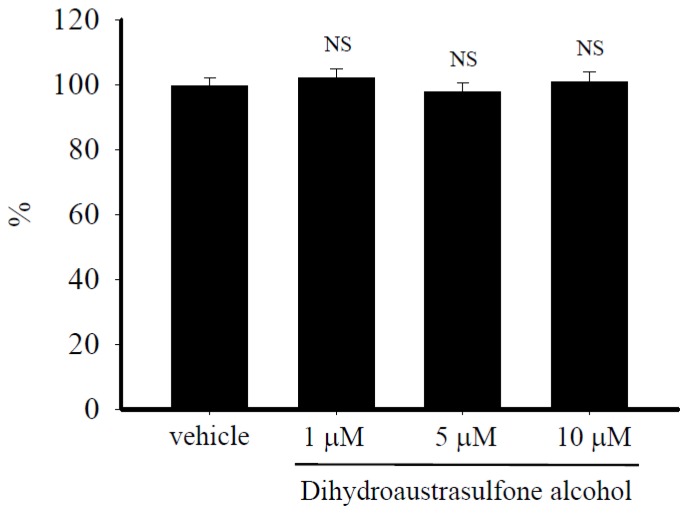
Effects of dihydroaustrasulfone alcohol on the viability of HASMCs. Quiescent HASMCs were treated with vehicle (0.1% DMSO) only or dihydroaustrasulfone alcohol (1, 5 or 10 µM) for 24 h. MTT was added into the culture medium for quantification of living cells. Mitochondrial dehydrogenases metabolize MTT, producing a purple formazan dye that can be measured using an ELISA plate reader scanning at a wavelength of 550 nm. Data are expressed as percentages relative to control (vehicle only) values. Results are the mean ± SEM of three independent experiments. NS indicates that the difference between the values is not statistically significant.

To evaluate the possibility that inhibition of human aortic smooth muscle cells (HASMCs) proliferation by dihydroaustrasulfone alcohol might be due to an effect on cell viability, the 3-(4,5-dimethylthiazol-2-yl)-2,5-diphenyltetrazolium bromide (MTT) viability assay was performed. Cell viability was not affected when HASMCs were treated with up to 10 µM dihydroaustrasulfone alcohol for 24 h (Figure 2). These results indicate that dihydroaustrasulfone alcohol is not cytotoxic for HASMCs and that it suppresses PDGF-induced proliferation of HASMCs without inducing cell death.

### 2.3. Dihydroaustrasulfone Alcohol Suppress the PDGF-Induced Migration of HASMCs

**Figure 3:**
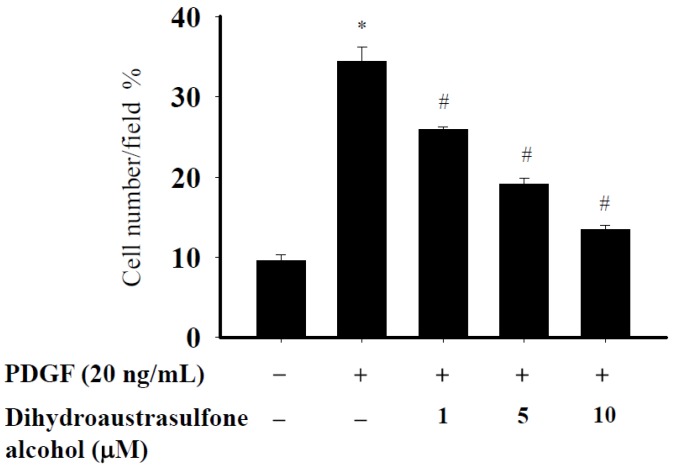
Dihydroaustrasulfone alcohol inhibits PDGF-induced migration of HASMCs. HASMC migration assays were performed using a modified Boyden chamber. Cells were seeded at 4 × 10^4^ cells per well in the upper chamber and pretreated with different concentrations of dihydroaustrasulfone alcohol. PDGF (20 ng/mL) was added to the lower chamber as a chemoattractant, and migration was allowed to proceed for 16 h before measurement. Data are expressed as the mean ± SEM of at least three independent experiments. * *p* < 0.05 *versus* control, ^#^ *p* < 0.05 *versus* PDGF alone.

To determine the effect of dihydroaustrasulfone alcohol on PDGF-induced migration, a modified Boyden chamber chemotaxis assay was used. Serum-starved HASMCs were treated with various concentrations of dihydroaustrasulfone alcohol in the upper side of each chamber. Cell migration was induced by adding PDGF (20 ng/mL) to the lower chamber. Dihydroaustrasulfone alcohol potently inhibited PDGF-induced migration of HASMCs in a dose-dependent manner (Figure 3).

### 2.4. Dihydroaustrasulfone Alcohol Affects Expression of Cell Cycle Regulatory Proteins in HASMCs

**Figure 4:**
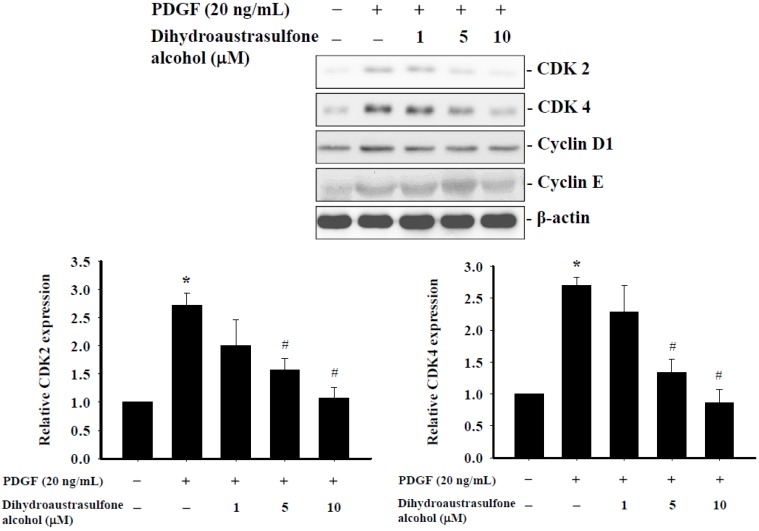

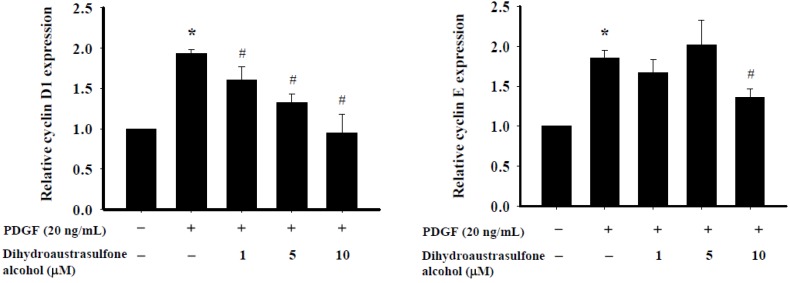
Effects of dihydroaustrasulfone alcohol on the expression of cell cycle regulatory proteins in HASMCs. Serum-starved HASMCs were stimulated with PDGF (20 ng/mL) for 24 h after pretreatment with different concentrations (1, 5 or 10 µM) of dihydroaustrasulfone alcohol. The expression of CDK2, CDK4, cyclin D1 and cyclin E was determined by Western blot analysis. Results were quantified using densitometry and normalized with respect to β-actin. Data are the means ± SEM of at least three independent experiments. * *p* < 0.05 *versus* control, ^#^ *p* < 0.05 *versus* PDGF alone.

The effects of dihydroaustrasulfone alcohol treatment on the expression of cell cycle regulatory proteins in HASMCs were examined. In the absence of dihydroaustrasulfone alcohol, the expression levels of cyclin-dependent kinases (CDK) 2, CDK4, cyclin D1 and cyclin E were significantly increased by PDGF stimulation compared with the untreated control cells. However, pretreatment with 5 µM or higher concentrations of dihydroaustrasulfone alcohol suppressed the PDGF-induced CDK2, CDK4, cyclin D1 and cyclin E upregulation (Figure 4). These findings provide evidence that the inhibitory effect of dihydroaustrasulfone alcohol on the proliferation of HASMCs is associated with cell cycle inhibition.

### 2.5. Dihydroaustrasulfone Alcohol Differentially Affects PDGF-Induced Phosphorylation of Extracellular Signal-Regulated Kinase 1/2 and Akt

**Figure 5:**
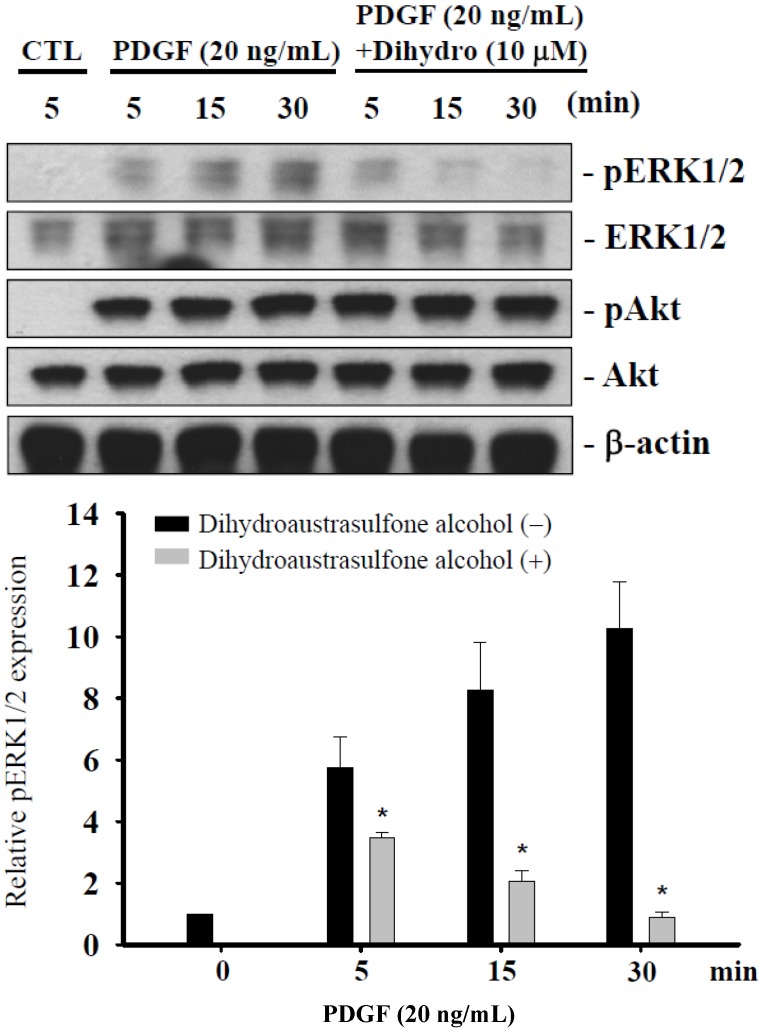
Effects of dihydroaustrasulfone alcohol on PDGF-induced ERK1/2 and Akt phosphorylation. Serum-starved HASMCs were stimulated with PDGF (20 ng/mL) for the indicated time periods, with or without 1 h pretreatment with 10 μM dihydroaustrasulfone alcohol (Dihydro). The cells were lysed, and the phosphorylation of ERK1/2 and Akt was determined using Western blotting. Data are the means ± SEM of at least three independent experiments. * *p* < 0.05 compared with the matching pair.

In mammalian cells, mitogen-activated protein kinase (MAPK) signaling pathways have important roles in the regulation of a variety of cellular functions, including proliferation, migration and survival [16,17]. MAPK and phosphatidylinositol 3-kinase (PI3K)/Akt cascades are activated by multiple extracellular stimuli, such as growth factors [18,19]. ERK is a member of the MAPK superfamily. ERK is activated by mitogenic stimuli and has a key role in the regulation of cellular proliferation and differentiation [20]. MAPK and the PI3K/Akt pathway are essential for cellular proliferation in response to many growth factors, and therefore, we investigated the effect of dihydroaustrasulfone alcohol on the phosphorylation of ERK1/2 and Akt. Treatment of cells with PDGF significantly increased phosphorylation of both of ERK1/2 and Akt within 5 min. Pretreatment with dihydroaustrasulfone alcohol significantly inhibited the phosphorylation of ERK1/2, but not Akt (Figure 5).

### 2.6. Dihydroaustrasulfone Alcohol Inhibits PDGF-Stimulated HASMCs Proliferation via the ERK Signaling Pathway

**Figure 6:**
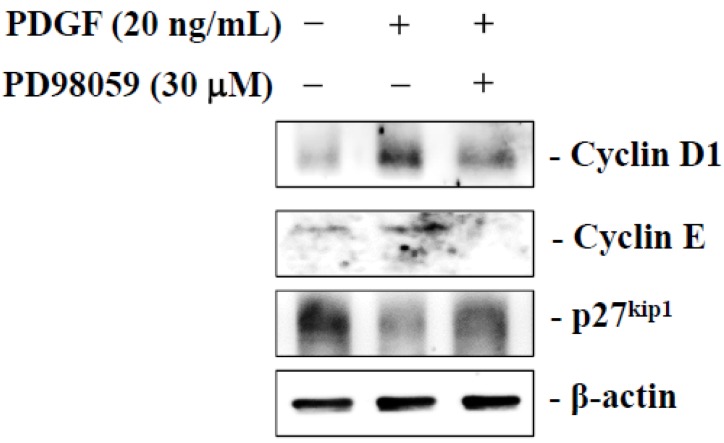
Effect of extracellular signal-regulated kinase (ERK) inhibition on platelet-derived growth factor (PDGF)-induced expression of cyclin D1, cyclin E and p27^kip1^. Serum-starved HASMCs were stimulated with PDGF in the presence of PD98059 (30 μM) for 24 h, and then, the expression levels of cyclin D1, cyclin E and 27^kip1^ were determined using Western blot analysis and compared with those of β-actin. The results represent at least three independent experiments.

To determine if dihydroaustrasulfone alcohol exerted an anti-proliferative effect by inhibiting ERK activity, we examined the effect of an ERK inhibitor on the expression of cyclin D1, cyclin E and p27^kip1^, a cyclin-dependent kinase inhibitor (CKIs). HASMCs treated with PDGF showed significantly increased cyclin D1 and cyclin E expression, but decreased p27^kip1^ expression. PD98059 (a highly selective inhibitor of ERK) reversed the PDGF-induced upregulation of cyclin D1 and cyclin E, while it downregulated p27^kip1^. These results suggest that dihydroaustrasulfone alcohol may suppress the PDGF-induced proliferation of HASMCs by inhibiting the activation of the ERK pathway (Figure 6).

### 2.7. Dihydroaustrasulfone Alcohol Modulates HASMCs Phenotype

**Figure 7:**
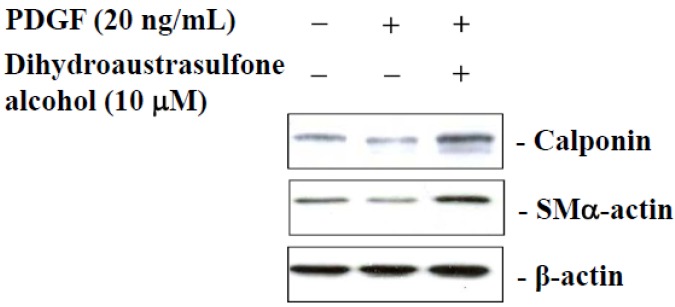
Dihydroaustrasulfone alcohol inhibits the PDGF-induced HASMC synthetic phenotype. HASMCs were pretreated with dihydroaustrasulfone alcohol (10 μM) 1 h prior to the 24-h PDGF treatment (20 ng/mL). The expression of calponin and SMα-actin was measured using Western blot analysis. The results represent at least three independent experiments.

The effects of dihydroaustrasulfone alcohol on the phenotypic modulation of HASMCs were investigated using a Western blot assay. The PDGF-treated cells showed a higher reduction in the expression of contractile marker proteins, such as calponin and SMα-actin, than the untreated control cells. Interestingly, dihydroaustrasulfone alcohol treatment reversed the phenotypic change induced by PDGF (Figure 7). These results demonstrate that dihydroaustrasulfone alcohol may alter the VSMC phenotype from a synthetic to contractile state.

### 2.8. Dihydroaustrasulfone Alcohol Reduces Neointimal Thickening by Modulating VSMC Phenotype in a Rat Model of Restenosis Induced by Carotid Artery Balloon Injury

**Figure 8:**
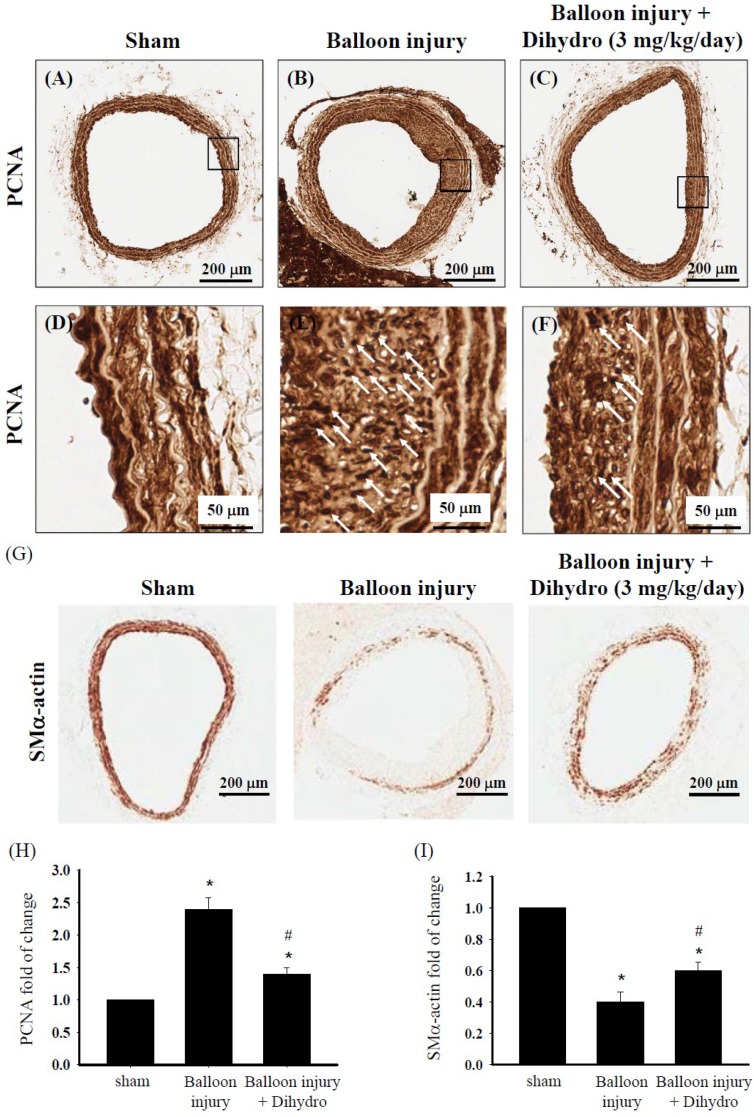
Dihydroaustrasulfone alcohol inhibits smooth muscle cell proliferation and alters smooth muscle cell phenotype after balloon catheter injury in rat carotid artery. Photomicrographs of PCNA (**A**–**F**) and SMα-actin (**G**) immunohistochemical staining of cross-sections of carotid artery taken three weeks after balloon injury in sham, balloon injury and dihydroaustrasulfone alcohol (Dihydro)-treated animals. Arrow heads indicate PCNA staining. Quantification of bar graphs showing PCAN and SMα-actin expression in arteries (**H**,**I**). Results are the means ± SEM of at least three independent experiments. * *p* < 0.05 *versus* sham group, ^#^ *p* < 0.05 *versus* balloon injury group.

Our *in vitro* results demonstrated that dihydroaustrasulfone alcohol significantly inhibits PDGF-induced proliferation, migration and synthetic phenotype formation in HASMCs. To determine whether these effects are reproduced *in vivo*, we performed studies on *in vivo* VSMC overproliferation in response to carotid artery balloon injury. The animals received dihydroaustrasulfone alcohol treatment (3 mg/kg/day) by intraperitoneal injection from Day 10 to 21 after the operation. Immunohistochemical staining with anti-PCNA and anti-SMα-actin antibodies was performed on cross-sections of tissue from the site of injury of the carotid artery. The expression of the proliferation marker PCNA was more significantly decreased in the dihydroaustrasulfone alcohol-treated group than in the balloon injury group (Figure 8A–F,H). Next, we performed immunostaining of the contractile phenotype marker SMα-actin to determine the VSMC phenotypic status in the region of arterial injury. Three weeks after injury, SMα-actin expression at the injury site of the dihydroaustrasulfone alcohol-treated group increased more than that of the balloon injury group (Figure 8G,I). These results suggest that dihydroaustrasulfone alcohol suppresses rat VSMC overproliferation induced by balloon injury and alters the phenotype of VSMCs at the injury site from a pathophysiological synthetic phenotype to a physiological contractile phenotype.

## 3. Discussion

Many types of cells are involved in vascular wall development. VSMCs are one of the important cells in vassal walls, which maintain vascular hemostasis [21]. Abnormal proliferation, migration and phenotypic modulation of VSMCs contributed to neointima formation during atherosclerosis and post-angioplasty-induced restenosis [22]. To date, treatment options for atherosclerosis and restenosis induced by abnormal VSMCs proliferation and migration, as well as phenotypic modulation are still limited. Therefore, the development of alternative therapeutics strategies is important. In our previous study, we showed that dihydroaustrasulfone alcohol has anti-neointimal thickening effects *in vivo* [11]. However, the detailed mechanism underlying the action of dihydroaustrasulfone alcohol on VSMCs remains unclear. In the present study, we investigated the inhibitory effect of dihydroaustrasulfone alcohol on PDGF-induced VSMC proliferation, migration and phenotypic modulation and clarified its underlying mechanisms. We found that dihydroaustrasulfone alcohol inhibits PDGF-induced proliferation and migration in cultured HASMCs dose-dependently without inducing cell death. Treatment with dihydroaustrasulfone alcohol inhibited cell-cycle progress by blocking G1 to S transition, and this was associated with the downregulation of cell cycle regulatory proteins. We also found that the antiproliferation effects of dihydroaustrasulfone alcohol were mediated by inhibition of the ERK pathway. Moreover, dihydroaustrasulfone alcohol modulated the VSMC phenotype from a synthetic to contractile state *in vivo* and *in vitro*. Therefore, our results indicate that the cellular effects of dihydroaustrasulfone alcohol on PDGF-induced HASMCs proliferation, migration and phenotypic modulation are highly relevant *in vivo*.

In the present study, we found that dihydroaustrasulfone alcohol inhibited PDGF-induced proliferation and migration by inhibiting phosphorylation of ERK1/2, but not Akt. Previous studies demonstrated that ERK is involved in cell growth, proliferation and differentiation, but activation of PI3K/Akt pathway is involved in the regulation of cell survival and anti-apoptosis [23,24,25,26]. This notion was supported by a recent study by Lim *et al.*, showing that peroxisome proliferator-activated receptor (PPAR) delta agonist inhibits rat vascular smooth muscle cell proliferation and migration by significantly inhibiting phosphorylation of ERK1/2, but not of Akt [27]. In order to examine whether the ERK signaling pathway was involved in the anti-proliferation effect of dihydroaustrasulfone alcohol, we used the highly-selective ERK inhibitor PD98059 to block ERK activity. We found that inhibition of ERK activity reversed the PDGF-induced upregulation of cell cycle regulatory proteins and downregulation of CDK inhibitor p27^kip1^. Therefore, these results indicate that the inhibitory effect of dihydroaustrasulfone alcohol on the proliferation of HASMCs is mediated through the ERK-dependent pathway.

A previous study reported that the monocyte chemoattractant protein-1 (MCP-1) and interleukin-8 (IL-8) are the markers of SMCs activation [28]. In the rat carotid artery balloon catheter injury model, the VSMCs continued to express high levels of MCP-1 [29]. MCP-1 and IL-8 are mitogens that induce proliferation of VSMCs [30,31]. A previous study has shown that dihydroaustrasulfone alcohol has anti-inflammatory property [11]. Therefore, we speculated that dihydroaustrasulfone alcohol inhibits the cell proliferation through inhibition of proinflammatory factors and associated signaling pathways. We will exam whether the anti-proliferation effect of dihydroaustrasulfone alcohol may be through inhibition of proinflammatory factors, such as MCP-1 and IL-8, in the injury site of VSMCs in the future.

The phenotypic switching of VSMCs in response to injury is a critical process for atherosclerosis progression and neointima formation [32,33]. However, the mechanisms involved in SMCs phenotype regulation have not been fully elucidated. An important finding of the present study is that we have characterized a novel mechanism by which dihydroaustrasulfone alcohol regulates the conversion of human VSMC phenotype from a synthetic to contractile state *in vitro* and *in vivo*. Our *in vivo* results also suggest that dihydroaustrasulfone alcohol may prevent neointimal thickening by modulating the VSMC phenotype. Previous studies demonstrated that transforming growth factor beta 1 (TGF-β1) was involved in modulating the SMCs contractile phenotype [34,35]. In our work, we observed that TGF-β1 was upregulated by dihydroaustrasulfone alcohol in macrophage (data not shown). Future studies will be required to elucidate the cellular mechanisms involved in the anti-synthetic phenotype conversion of smooth muscle cells by dihydroaustrasulfone alcohol.

In this study, we showed that dihydroaustrasulfone alcohol significantly and dose-dependently inhibited PDGF-induced migration in HASMCs. There is growing evidence indicating that dihydroaustrasulfone alcohol inhibits cell migration in human non-small cell lung cancer A549 cells and endothelial cells by inhibiting the activities and expression of matrix metalloproteinase (MMP)-2 and MMP-9, thereby suppressing lung cancer cell growth and angiogenesis [12,36]. Our *in vitro* and *in vivo* data show that dihydroaustrasulfone alcohol inhibits PDGF-induced migration of HASMCs and balloon injury-induced neointimal thickening in rats. Therefore, the detailed mechanisms of the anti-migration effects of dihydroaustrasulfone alcohol in VSMCs should be elucidated in future studies.

## 4. Experimental Section

### 4.1. Materials

Dihydroaustrasulfone alcohol was synthesized from its precursor, austrasulfone, using previously described procedures, which include its structure [11,12]. PDGF was purchased from R & D Systems, Inc. (Minneapolis, MN, USA). Mouse monoclonal antibodies against CDK2, CDK4, cyclin D1, cyclin E, ERK1/2, phospho-ERK1/2, as well as rabbit polyclonal antibodies against p27^kip1^, Akt and phospho-Akt (Ser473) were purchased from Cell Signaling Technology (Beverly, MA, USA). Mouse monoclonal antibodies against calponin and SMα-actin were purchased from Sigma (St Louis, Mo, USA).

### 4.2. Cell Culture and Drug Treatment

HASMCs were purchased from Cambrex Corporation (East Rutherford, NJ, USA) and maintained in F12K medium (Gibco) supplemented with 10% fetal bovine serum (FBS), 100 U/mL penicillin and 100 mg/mL streptomycin. Cells between Passages 4 and 6 were used in the assays. When cells reached 70%–80% confluence, they were serum-starved in F12K medium containing 0.1% FBS for 24 h. After starvation, the cells were pretreated with dihydroaustrasulfone alcohol (1, 5, or 10 μM) for 1 h and then stimulated by the addition of PDGF.

### 4.3. BrdU Incorporation Assay

Cells were seeded at 4 × 10^4^ cells/well in 96-well plates and treated with dihydroaustrasulfone alcohol and/or PDGF. Treated cells were incubated with BrdU antibody, and the cellular proliferation was determined by the BrdU incorporation assay using a commercial enzyme-linked immunosorbent assay (ELISA) kit according to the manufacturer’s instructions (Roche, Penzberg, Germany).

### 4.4. Flow Cytometry Analysis

Flow cytometry was used to analyze cell cycle distribution. In brief, cells were harvested in PBS containing 2 mM ethylenediaminetetraacetic acid (EDTA), washed once with PBS and fixed in cold 70% ethanol for 30 min. The fixed cells were washed in PBS and permeabilized by incubation with 0.2% Tween 20 and 1 mg/mL RNase A in PBS for another 30 min. They were then washed in PBS and stained with 50 μg/mL of propidium iodide (Roche, Basel, Switzerland). Stained cells were analyzed using a fluorescence activated cell sorter (FACS Calibur; Becton Dickinson, Franklin Lakes, NJ, USA), and the data were analyzed using a mod-fit cell cycle analysis program.

### 4.5. Cell Viability Assay

Cell viability was determined by the 3-(4,5-dimethylthiazol-2-yl)-2,5-diphenyltetrazolium bromide (MTT) assay. Briefly, HASMCs were seeded in 96-well plates with 4 × 10^4^ cells/well in culture medium. The cells were treated with various concentrations of dihydroaustrasulfone alcohol (1, 5 or 10 µM) for 24 h. After washing with culture medium, MTT was added into each well to a final concentration of 5 mg/mL for quantification of living, metabolically-active cells, and the plates were incubated at 37 °C for 3 h. Finally, the medium was removed, and 50 μL dimethyl sulfoxide (DMSO) were added. Mitochondrial dehydrogenases metabolize MTT to form a purple formazan dye, which was measured by using the ELISA reader with the absorbance at 550 nm.

### 4.6. Cell Migration Assay

VSMC migration was examined by using a modified Boyden chamber, as previously described [37]. Briefly, HASMCs (4 × 10^4^ cells/well) were seeded onto the upper surface of an 8-µm pore size chamber (Costar Inc., Corning, NY, USA) in serum-free medium containing 0.2% bovine albumin serum (BSA) and then pretreated with various concentrations of dihydroaustrasulfone alcohol (1, 5 or 10 µM). PDGF (20 ng/mL) was added only in the lower chamber, and cells were then incubated at 37 °C in air containing 5% CO_2_. After 16 h, cells that had not migrated were removed from the upper chamber, and the cells that had migrated to the lower surface of the filter were fixed with methanol for 10 min at 4 °C. Migrated cells were then stained and counted from at least five fields for each well, using a microscope (100× magnification).

### 4.7. Western Blot Analysis

Cultured HASMCs were collected by scraping and lysed using a buffer containing 1% NP-40, 0.5% sodium deoxycholate, 0.1% sodium dodecyl sulfate (SDS) and a protease inhibitor mixture (PMSF, aprotinin and sodium orthovanadate). The total cell lysate was separated by SDS-PAGE and transferred onto a nitrocellulose membrane. The membrane was then incubated with the designated antibodies. Immunodetection was performed by using the Western-Light chemiluminescent detection system (Applied Biosystems, Foster City, CA, USA).

### 4.8. Rat Balloon Injury Model

The balloon-induced carotid artery injury model used male Sprague-Dawley rats that weighed between 250–300 g and was developed as previously described [38,39]. The animals were separated into three groups: sham, balloon injury with vehicle (DMSO) treatment and dihydroaustrasulfone alcohol (3 mg/kg/day)-treated injury groups. The animals were sacrificed after 3 weeks, and the right and left common carotid arteries were dissected out. Tissue sections were prepared and immunohistochemically stained as described in our previous studies [9,40]. The use of animals conformed to the guidelines in the Care and Use of Animals of the American Physiology Society, and the study was approved by the National Sun Yat-sen University Animal Care and Use Committee (No. 10117).

### 4.9. Statistical Analysis

Results are expressed as the mean ± standard error of the mean (SEM) of at least three independent experiments. Statistical analysis was performed using Student’s *t*-tests. *p*-values <0.05 were considered statistically significant.

## 5. Conclusions

In summary, our results demonstrate that dihydroaustrasulfone alcohol inhibits abnormal proliferation and migration of VSMCs at the injury site in a rat model of atherosclerosis and restenosis. Moreover, dihydroaustrasulfone alcohol also modulated the phenotypic conversion of VSMCs from a pathophysiological synthetic phenotype to a physiological contractile state *in vitro* and *in vivo*. Therefore, these findings illustrate that dihydroaustrasulfone alcohol may be a potentially important therapeutic strategy for the treatment of vascular occlusive disease.

## References

[1] Ross R. (1993). The pathogenesis of atherosclerosis: A perspective for the 1990s. Nature.

[2] Owens G.K., Kumar M.S., Wamhoff B.R. (2004). Molecular regulation of vascular smooth muscle cell differentiation in development and disease. Physiol. Rev..

[3] Schwartz S.M. (1997). Smooth muscle migration in atherosclerosis and restenosis. J. Clin. Invest..

[4] Majesky M.W., Reidy M.A., Bowen-Pope D.F., Hart C.E., Wilcox J.N., Schwartz S.M. (1990). PDGF ligand and receptor gene expression during repair of arterial injury. J. Cell Biol..

[5] Miano J.M., Vlasic N., Tota R.R., Stemerman M.B. (1993). Smooth muscle cell immediate-early gene and growth factor activation follows vascular injury. A putative *in vivo* mechanism for autocrine growth. Arterioscler. Thromb..

[6] Ross R., Bowen-Pope D.F., Raines E.W. (1990). Platelet-derived growth factor and its role in health and disease. Philos. Trans. R. Soc. Lond. B Biol. Sci..

[7] Jawien A., Bowen-Pope D.F., Lindner V., Schwartz S.M., Clowes A.W. (1992). Platelet-derived growth factor promotes smooth muscle migration and intimal thickening in a rat model of balloon angioplasty. J. Clin. Investig..

[8] Libby P., Warner S.J., Salomon R.N., Birinyi L.K. (1988). Production of platelet-derived growth factor-like mitogen by smooth muscle cells from human atheroma. N. Engl. J. Med..

[9] Jean Y.H., Chen W.F., Duh C.Y., Huang S.Y., Hsu C.H., Lin C.S., Sung C.S., Chen I.M., Wen Z.H. (2008). Inducible nitric oxide synthase and cyclooxygenase-2 participate in anti-inflammatory and analgesic effects of the natural marine compound lemnalol from Formosan soft coral Lemnalia cervicorni. Eur. J. Pharmacol..

[10] Jean Y.H., Chen W.F., Sung C.S., Duh C.Y., Huang S.Y., Lin C.S., Tai M.H., Tzeng S.F., Wen Z.H. (2009). Capnellene, a natural marine compound derived from soft coral, attenuates chronic constriction injury-induced neuropathic pain in rats. Br. J. Pharmacol..

[11] Wen Z.H., Chao C.H., Wu M.H., Sheu J.H. (2010). A neuroprotective sulfone of marine origin and the *in vivo* anti-inflammatory activity of an analogue. Eur. J. Med. Chem..

[12] Chen S.C., Chien Y.C., Pan C.H., Sheu J.H., Chen C.Y., Wu C.H. (2014). Inhibitory effect of dihydroaustrasulfone alcohol on the migration of human non-small cell lung carcinoma A549 cells and the antitumor effect on a Lewis lung carcinoma-bearing tumor model in C57BL/6J mice. Mar. Drugs.

[13] Schwartz R.S., Murphy J.G., Edwards W.D., Camrud A.R., Vliestra R.E., Holmes D.R. (1990). Restenosis after balloon angioplasty. A practical proliferative model in porcine coronary arteries. Circulation.

[14] Ferns G.A., Avades T.Y. (2000). The mechanisms of coronary restenosis: Insights from experimental models. Int. J. Exp. Pathol..

[15] Leimgruber P.P., Roubin G.S., Hollman J., Cotsonis G.A., Meier B., Douglas J.S., King S.B., Gruentzig A.R. (1986). Restenosis after successful coronary angioplasty in patients with single-vessel disease. Circulation.

[16] Muslin A.J. (2008). MAPK signalling in cardiovascular health and disease: Molecular mechanisms and therapeutic targets. Clin. Sci. (Lond.).

[17] Zhan Y., Kim S., Izumi Y., Izumiya Y., Nakao T., Miyazaki H., Iwao H. (2003). Role of JNK, p38, and ERK in platelet-derived growth factor-induced vascular proliferation, migration, and gene expression. Arterioscler. Thromb. Vasc. Biol..

[18] Johnson G.L., Lapadat R. (2002). Mitogen-activated protein kinase pathways mediated by ERK, JNK, and p38 protein kinases. Science.

[19] Cantley L.C. (2002). The phosphoinositide 3-kinase pathway. Science.

[20] Force T., Bonventre J.V. (1998). Growth factors and mitogen-activated protein kinases. Hypertension.

[21] Ross R. (1999). Atherosclerosis-an inflammatory disease. N. Engl. J. Med..

[22] Ross R. (1990). Mechanisms of atherosclerosis—A review. Adv. Nephrol. Necker Hosp..

[23] Crews C.M., Alessandrini A., Erikson R.L. (1992). The primary structure of MEK, a protein kinase that phosphorylates the ERK gene product. Science.

[24] Hemmings B.A. (1997). Akt signaling: Linking membrane events to life and death decisions. Science.

[25] Franke T.F., Kaplan D.R., Cantley L.C. (1997). PI3K: Downstream AKTion blocks apoptosis. Cell.

[26] Kulik G., Klippel A., Weber M.J. (1997). Antiapoptotic signalling by the insulin-like growth factor I receptor, phosphatidylinositol 3-kinase, and Akt. Mol. Cell. Biol..

[27] Lim H.J., Lee S., Park J.H., Lee K.S., Choi H.E., Chung K.S., Lee H.H., Park H.Y. (2009). PPAR delta agonist L-165041 inhibits rat vascular smooth muscle cell proliferation and migration via inhibition of cell cycle. Atherosclerosis.

[28] Rainger G.E., Nash G.B. (2001). Cellular pathology of atherosclerosis: Smooth muscle cells prime co-cultured endothelial cells for enhanced leukocyte adhesion. Circ. Res..

[29] Landry D.B., Couper L.L., Bryant S.R., Lindner V. (1997). Activation of the NF-kappa B and I kappa B system in smooth muscle cells after rat arterial injury. Induction of VCAM-1 and MCP-1. Am. J. Pathol..

[30] Porreca E., Di Febbo C., Reale M., Castellani M.L., Baccante G., Barbacane R., Conti P., Cuccurullo F., Poggi A. (1997). Monocyte chemotactic protein 1 (MCP-1) is a mitogen for cultured rat vascular smooth muscle cells. J. Vasc. Res..

[31] Yue T.L., Wang X., Sung C.P., Olson B., McKenna P.J., Gu J.L., Feuerstein G.Z. (1994). Interleukin-8—A mitogen and chemoattractant for vascular smooth muscle cells. Circ. Res..

[32] Dzau V.J., Braun-Dullaeus R.C., Sedding D.G. (2002). Vascular proliferation and atherosclerosis: New perspectives and therapeutic strategies. Nat. Med..

[33] Rensen S.S., Doevendans P.A., van Eys G.J. (2007). Regulation and characteristics of vascular smooth muscle cell phenotypic diversity. Neth. Heart J..

[34] Tang Y., Yang X., Friesel R.E., Vary C.P., Liaw L. (2011). Mechanisms of TGF-β-induced differentiation in human vascular smooth muscle cells. J. Vasc. Res..

[35] Wang X., Hu G., Betts C., Harmon E.Y., Keller R.S., Van De Water L., Zhou J. (2011). Transforming growth factor-β1-induced transcript 1 protein, a novel marker for smooth muscle contractile phenotype, is regulated by serum response factor/myocardin protein. J. Biol. Chem..

[36] Lin S.W., Huang S.C., Kuo H.M., Chen C.H., Ma Y.L., Chu T.H., Bee Y.S., Wang E.M., Wu C.Y., Sung P.J. (2015). Coral-derived compound WA-25 inhibits angiogenesis by attenuating the VEGF/VEGFR2 signaling pathway. Mar. Drugs.

[37] Redmond E.M., Cahill P.A., Hirsch M., Wang Y.N., Sitzmann J.V., Okada S.S. (1999). Effect of pulse pressure on vascular smooth muscle cell migration: The role of urokinase and matrix metalloproteinase. Thromb. Haemost..

[38] Berger M., Rubinraut E., Barshack I., Roth A., Keren G., George J. (2004). Zinc reduces intimal hyperplasia in the rat carotid injury model. Atherosclerosis.

[39] Chen J.H., Wu C.C., Hsiao G., Yen M.H. (2003). Magnolol induces apoptosis in vascular smooth muscle. Naunyn Schmiedebergs Arch. Pharmacol..

[40] Huang S.Y., Chen N.F., Chen W.F., Hung H.C., Lee H.P., Lin Y.Y., Wang H.M., Sung P.J., Sheu J.H., Wen Z.H. (2012). Sinularin from indigenous soft coral attenuates nociceptive responses and spinal neuroinflammation in carrageenan-induced inflammatory rat model. Mar. Drugs.

